# TIPE1 Promotes Cervical Cancer Cell Chemoresistance to Cisplatin in a Wild-Type p53-Dependent Manner

**DOI:** 10.3389/fonc.2020.593615

**Published:** 2021-01-15

**Authors:** Jie Jiang, Li Gao, Yongting Lan, Yang Wang, Peiqing Zhao

**Affiliations:** ^1^ Department of Clinical Laboratory, Yantai Affiliated Hospital of Binzhou Medical University, Yantai, China; ^2^ Department of Stomatology, Zibo Central Hospital, Shandong University, Zibo, China; ^3^ Center of Translational Medicine, Zibo Central Hospital, Shandong University, Zibo, China; ^4^ Department of Clinical Laboratory, Huantai County People’s Hospital, Zibo, China

**Keywords:** apoptosis, p53, cervical cancer, chemoresistance, TIPE1

## Abstract

Previous studies have revealed that TIPE1 serves as a tumor suppressor gene in several tumor types. However, we demonstrated that TIPE1 can promote cervical cancer proliferation by suppressing p53 activity. Here, we showed that TIPE1 inhibits cervical cancer cell apoptosis both *in vivo* and *in vitro*. Mechanistically, we revealed that TIPE1 facilitates chemoresistance in a wild-type p53-dependent manner. The results indicated that TIPE1 is responsible for the transition from chemosensitivity to chemoresistance, and that it can serve as a promising target in cervical cancer chemotherapy.

## Introduction

Gynecological tumors, accounting for approximately 18% of all female cancers, affect approximately 100 million people worldwide each year, and cervical cancer is the most common female reproductive system tumor in developing countries (1). The treatment of advanced-stage cervical cancer is primarily concurrent chemoradiotherapy, and it has better outcomes than radiation therapy or surgery alone (2). However, the success of chemotherapy is limited mainly owing to the chemoresistance, especially in patients with tumors derived from epithelial tissues such as cervical cancer (3).

Chemoresistance is exhibited *via* a variety of distinct mechanisms. Notably, wild-type p53(wt-p53) plays important roles in the maintenance of DNA integrity in response to anticancer drugs (2). Interestingly, the effects of p53 on the acquisition of the multidrug resistance (MDR) phenotype depend on its status. Wt-p53 globally plays an inhibitory role in drug resistance, while mutant p53(mut-p53) has the reverse function and results in resistance to chemotherapy drugs (4).

Tumor necrosis factor α-induced protein 8 (TNFAIP8)-like protein 1 (TIPE1) belongs to the TIPE family. It was first identified as an apoptotic factor involved in the process of necroptosis. Knockdown of TIPE1 inhibits necroptosis and apoptosis in L929 cells and NIH3T3 cells. Recent studies also revealed that TIPE1 probably serves as a tumor suppressor gene in several tumor types (5). However, we recently showed that TIPE1 can promote cervical cancer proliferation by suppressing p53 activity (6). Thus, we asked whether TIPE1 affects the process of cervical cancer chemoresistance depending on p53, and whether TIPE1 participates in this process relies on the status of p53.

## Downregulation Of TIPE1 Leads to Increased Apoptosis in Chemoresistant Cervical Cancer

**Figure 1:**
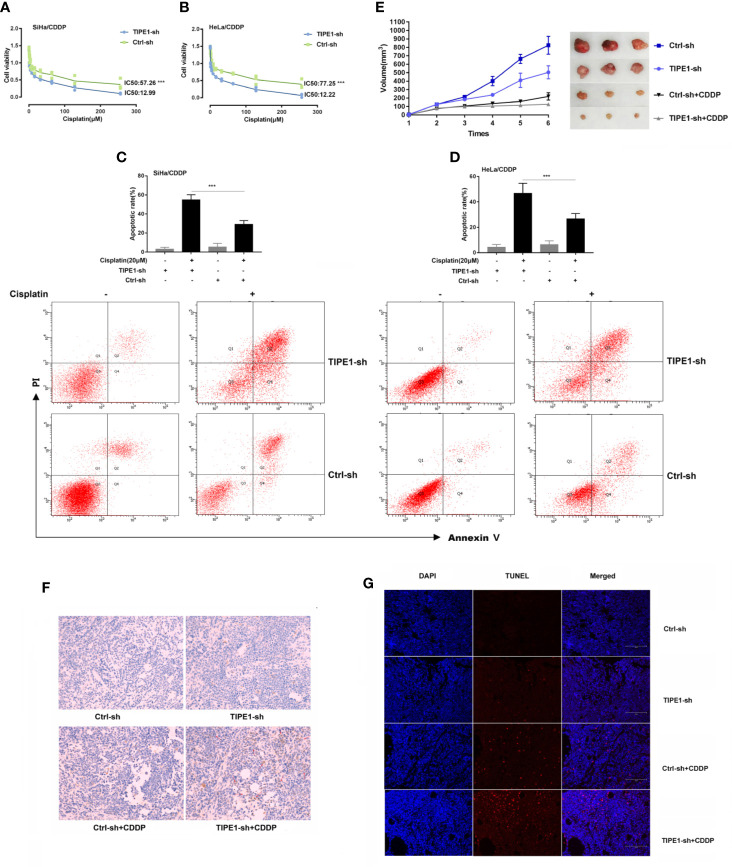
TIPE1 decreases CDDP sensitivity both *in vitro* and *in vivo.* **(A, B)** TIPE1-sh overexpression significantly increased CDDP-induced cytotoxicity in CDDP-resistant cervical cancer cells. **(C, D)** Overexpression of TIPE1-sh enhanced CDDP-induced apoptosis by flow cytometry. **(E)** Xenograft tumor models with SiHa/CDDP cells containing TIPE1-sh had an overall reduction in tumor volume compared to that in the control with or without CDDP treatment (CDDP 30 ug/kg). **(F)** TUNEL staining confirmed that TIPE1-sh increased CDDP*-*induced apoptosis. **(G)** TIPE1-sh increased the expression level of caspase-3, as detected by using an immunohistochemistry assay. Animal experiments were repeated twice with similar results and at least three mice were included in each group. Values represent means ± SD; for all panels, ***p < 0.001.

Here, we revealed that TIPE1 inhibits chemoresistant cervical cancer cell apoptosis both *in vivo* and *in vitro*. We first exposed cervical cancer cell lines to various doses of cisplatin (CDDP) to generate cisplatin-resistant cervical cancer cells (named as SiHa/CDDP and HeLa/CDDP) according to our previous report. We observed the expression of TIPE1 in cisplatin-resistant cervical cancer cell lines is increased compared to their matched sensitive cell lines (Data not shown). Next, the cell survival rate and IC50 in SiHa/CDDP and HeLa/CDDP cell lines transfected with or without TIPE1 siRNA (TIPE1-sh) were measured with increasing cisplatin treatment (from 0 to 250 μM). The results demonstrated that the cells transfected with control vectors (Ctrl-sh) were more prone to survive compared to the TIPE1-sh group in response to cisplatin (**Figures 1A, B**). For the apoptosis study, flow cytometry methods were used to investigate whether TIPE1 affects the apoptosis rate in cisplatin-resistant cell lines treated with cisplatin (20 μM). Not surprisingly, the results revealed that the apoptosis population was increased after TIPE1-sh overexpression (**Figures 1C, D**).

Given that TIPE1 has been substantiated as a positive inducer in cervical cancer chemoresistance, we further verified its role in chemoresistance *in vivo*. The nude mice were randomly divided into four groups. Two groups contained the TIPE1-sh vector, and the others contained the control vector (Ctrl-sh). Next, two groups, including one TIPE1-sh group and one control group, were treated with cisplatin (30 μg/kg) by intraperitoneal injection. The results demonstrated that mice implanted with cells transfected with TIPE1-sh developed smaller tumor volumes than the control group. More importantly, TIPE1 more significantly offset the reduction in tumor volume caused by chemotherapy drugs (**Figure 1E**). Furthermore, we confirmed that the groups transfected with the TIPE1-sh vector exhibited decreased resistance to cisplatin treatment as measured by the TUNEL method (**Figure 1F**), and had a decreased expression level of caspase-3 using an immunohistochemistry assay (**Figure 1G**).

## TIPE1 Enhances Cervical Cancer Chemoresistance in a Wild-Type P53-Dependent Manner

Previous studies have shown that TIPE1 can inhibit cell growth and induce cell death in several types of cancer. Supporting this, our group also found that TIPE1 inhibits breast cancer proliferation by inhibiting the ERK pathway (7). However, we recently demonstrated that TIPE1 significantly induces cell proliferation and tumor burden in cervical cancer (6). This TIPE1-mediated induction in cervical cancer cell growth was governed by inhibition of p53 activity. Similar to cervical cancer, our group also revealed that TIPE1 was upregulated in nasopharyngeal carcinoma, in which TIPE1 plays an important role in the induction of cell growth, proliferation and colony formation (8). Another study has shown that the expression of TIPE1 was upregulated in virus infection-related cancer cell lines (9). The occurrence of nasopharyngeal carcinoma is also closely related to Human Papilloma Virus (HPV) or Epstein-Barr Virus (EBV) infection. Therefore, we believe that TIPE1 may play a unique biological role in virus infection-related tumors.

**Figure 2:**
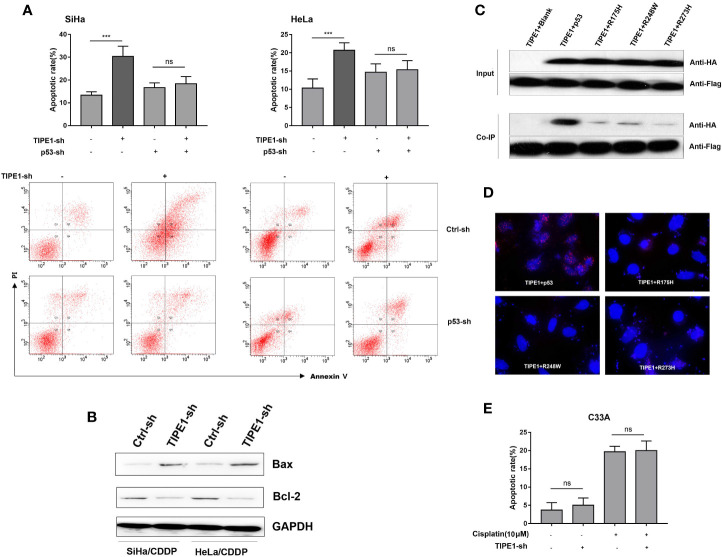
TIPE1 inhibits resistant cervical cancer cell apoptosis in a wt-p53-dependent manner. **(A)** TIPE1 inhibited SiHa/CDDP and HeLa/CDDP cells apoptosis in a p53-dependent manner. **(B)** Overexpression of TIPE1-sh enhances the expression of Bax and decreases Bcl-2 levels, as detected by using western blot analysis. Western blot **(C)** and Doulink methods **(D)** showing that TIPE1 primarily interacted with wt-p53. **(E)** TIPE1 did not induce apoptosis in cervical cancer cells containing mut-p53. These experiments were repeated at least three times. Values represent means ± SD; for all panels, ***p < 0.001, “ns” means no statistic.

Notably, only wt-p53 triggers increased apoptosis and cell cycle arrest-related genes in the cellular response to DNA damage induced by chemotherapy (10). Unlike other human malignancies that have a p53 mutation rate higher than 50%, human cervical cancer tissues generally exhibit wt-p53 (11). Our previous study revealed that TIPE1 interacted with p53 and thus suppressed its activity. This result preliminarily suggested that TIPE1 might promote resistance to cisplatin-induced apoptosis in a p53-dependent manner. To verify this hypothesis, we transfected TIPE1-sh into SiHa/CDDP and HeLa/CDDP cells and then detected cell apoptosis with a cisplatin administration (20 μM). The results showed that the cell apoptosis rate was upregulated with transfection of TIPE1-sh, but this effect was attenuated after p53 silencing (**Figure 2A**). Moreover, Bax levels (a target gene of p53) were also upregulated while BCL-2 was downregulated after transfection with TIPE1-sh (**Figure 2B**). This result preliminarily demonstrated that TIPE1 decreased the apoptosis rate in cisplatin-resistant cell lines by decreasing the expression of p53 target genes, such as Bax. Because approximately 10% of patients with cervical cancer express mut-p53, we investigated whether TIPE1 interacts with mut-p53 as well. Normally, only wt-p53 can effectively enhance the sensitivity of chemotherapy drugs (12). Thus, we constructed a series of mut-p53 vectors which including hot spot mut-p53 proteins (R175H, R248W, and R273H). SiHa cells were then co-transfected with TIPE1-Flag and wt-p53-HA or a series of mut-p53-HA expression plasmids. Interestingly, the result showed that mut-p53 proteins interact with TIPE1 to a lesser extent than wt-p53 (**Figure 2C**). Furthermore, we checked whether endogenous TIPE1 could interact with endogenous p53 using Doulink method, and the result showed the same phenomenon (**Figure 2D**). Because the effect of p53 on the acquisition of the multidrug resistance phenotype depends on its status, patients with cervical cancer who exhibit wt-p53 are notably different from patients with mut-p53 in terms of chemotherapy resistance. Finally, we transfected the TIPE1-sh vector into C33A cells (containing mutant p53^273C^), and then detected the apoptosis rate after treatment with cisplatin. The results showed that TIPE1 did not enhance chemoresistance in cervical cancer cells with mut-p53 (**Figure 2E**). These results demonstrated that TIPE1 promotes cervical cancer cells resistance to cisplatin-induced apoptosis in a wt-p53-dependent manner.

## Discussion

The TIPE family plays important roles in the regulation of cell death and the development of cancers. TIPE1 was mostly found to be antitumorigenic, and downregulated in most cancers (5). Here, we revealed that TIPE1 inhibits cervical cancer cell apoptosis both *in vivo* and *in vitro*. Mechanistically, we demonstrated that TIPE1 facilitates chemoresistance in a wt-p53-dependent manner. The results indicated that TIPE1 is responsible for the transition from chemosensitivity to chemoresistance, and that it can serve as a promising target in cervical cancer chemotherapy. Although we have demonstrated that TIPE1 can facilitate chemoresistance in cervical cancer cells in a wt-p53-dependent manner, more extensive studies are required to unveil the actual roles of TIPE1 in virus infection-related cancers.

## Data Availability Statement

The raw data supporting the conclusions of this article will be made available by the authors, without undue reservation.

## Ethics Statement

The animal study was reviewed and approved by Shandong University ethics committee.

## Author Contributions

PZ and JJ contributed to the conception and design of the study. LG, YL, and YW contributed to the sample collection and data acquisition. JJ and YL executed the study. PZ drafted the manuscript and revised the manuscript. All authors contributed to the article and approved the submitted version.

## Funding

This work was supported by the Natural Science Foundation of China (grant nos. 81972002), the Natural Science Foundation of Shandong Province (ZR2019MH099), and the Medicine and Health Science Technology Development Projects of Shandong Province (grant nos. 2018WS534/2016WS0748).

## Conflict of Interest

The authors declare that the research was conducted in the absence of any commercial or financial relationships that could be construed as a potential conflict of interest.
